# Electricity in the Suppression of the Lacteal Secretion

**Published:** 1857-09

**Authors:** 


*Electricity in the Suppression of the Lactial Secretion.*—M. Bicqurel, in a late communication to the *Société Médicale des Hôpitax de Paris*, has made some remarks upon the influence of electricity in restoring the secretion of milk. His attention was called to the subject by a case related to him by M. Aubert, who had employed electricity in the case of a young woman, whose milk had been suppressed in consequence of a double pneumonia. The electricity was applied to the breast by means of moist excitors, and after four applications, each lasting twenty minutes, the lactial secretion was completely restored—*British and Foreign Medico-Chirurgical Review.*

